# A sequence motif enriched in regions bound by the *Drosophila *dosage compensation complex

**DOI:** 10.1186/1471-2164-11-169

**Published:** 2010-03-12

**Authors:** Miguel Gallach, Vicente Arnau, Rodrigo Aldecoa, Ignacio Marín

**Affiliations:** 1 Department of Biology, University of Texas at Arlington, Arlington, Texas, USA; 2 Departamento de Informática, Universidad de Valencia, Valencia, Spain; 3 Instituto de Biomedicina de Valencia, Consejo Superior de Investigaciones Científicas (IBV-CSIC), Valencia, Spain

## Abstract

**Background:**

In *Drosophila melanogaster*, dosage compensation is mediated by the action of the dosage compensation complex (DCC). How the DCC recognizes the fly X chromosome is still poorly understood. Characteristic sequence signatures at all DCC binding sites have not hitherto been found.

**Results:**

In this study, we compare the known binding sites of the DCC with oligonucleotide profiles that measure the specificity of the sequences of the *D. melanogaster *X chromosome. We show that the X chromosome regions bound by the DCC are enriched for a particular type of short, repetitive sequences. Their distribution suggests that these sequences contribute to chromosome recognition, the generation of DCC binding sites and/or the local spreading of the complex. Comparative data indicate that the same sequences may be involved in dosage compensation in other *Drosophila *species.

**Conclusions:**

These results offer an explanation for the wild-type binding of the DCC along the *Drosophila *X chromosome, contribute to delineate the forces leading to the establishment of dosage compensation and suggest new experimental approaches to understand the precise biochemical features of the dosage compensation system.

## Background

In *Drosophila*, dosage compensation occurs by hypertranscription of the genes of the single X chromosome in males, leading to a level of expression similar to that found for the copies of those same genes located on the two female X chromosomes [1-3]. This hypertranscription is controlled by a ribonucleoprotein complex, known as Dosage Compensation Complex (DCC; a. k. a. MSL complex, compensasome), which includes at least five proteins, encoded by the *male-specific lethal *(MSL) genes [4-8], and two non-coding RNAs, derived from the *roX1 *and *roX2 *genes [9-11]. The DCC, functional only in males, modifies the chromatin structure of the X chromosome by altering its pattern of histone acetylation [12,13]. Immunostaining with antibodies against MSL proteins demonstrated that the DCC complex specifically recognizes hundreds of sites along the male X chromosome [14-20]. How this specificity is achieved is still poorly understood. Data obtained by hybridizing chromatin immunoprecipitates obtained from regions bound by the DCC complex to genomic tiling arrays (ChIP-chip) have led to the precise characterization of many binding sites ([21,22]; see also ref. [23]). So far, however, a common sequence motif shared by those regions has not been described. Just a slight enrichment for some very short sequences has been detected in those experiments, as well as with related techniques [21-25]. Partial DCCs are still generated in flies that are mutant for some of the genes encoding proteins of the complex. These incomplete DCCs are also able to recognize the X chromosome, but only in a limited number of places, first described by cytological analyses [16]. These places were suggested to be "entry sites", high-affinity binding sites from which the DCC would epigenetically spread to the rest of the chromosome [26]. Later, convincing evidence was obtained against generalized spreading from the few cytologically characterized sites as the only determinant for the wild-type pattern of DCC binding [27,28]. Recent data support however that the complex indeed has a higher affinity for those sites than for the rest of the X chromosome [29] and that these sites are more abundant than the cytological data suggested [29,30]. Therefore, they may contribute to spreading at a local scale [30-32]. A sequence motif, containing several GA/TC dinucleotides, has been found to be enriched in high affinity binding sites of the DCC ([29,30]; we will refer to this motif throughout the text as [GA/TC]_n_).

All these results are compatible with a genetic model in which the X chromosome is enriched for two different types of sequences. One kind of sequences, characterized by the (GA/TC)_n _motif, would be required for the complex to bind the high affinity sites. The other type, which is yet to be characterized, would be needed to achieve wild-type pattern of DCC binding. However, the characterization of epigenetic marks, often associated to gene transcription, which contribute to DCC binding, and the discovery of acquisition of DCC binding by some transcribed autosomal genes transposed to the X chromosome, has led to an alternative model: the DCCs would spread from the high affinity sites largely or even exclusively by following epigenetic signals [31,33-36]. These models are not in contradiction and formulating a combined model is possible (summarized in refs. [37,38]). On one hand, the *Drosophila *X chromosome may contain a large number of sites, with characteristic sequences able to attract the DCC with variable strengths. On the other hand, transcriptional status and epigenetic signals may influence the binding and/or the spreading of the complex. Obviously, this hybrid model could be possible only if a certain type of sequences is found to be enriched at all DCC binding sites and not only at the high affinity ones.

We recently developed a new type of DNA sequence analysis, called oligonucleotide profiling, which allows for the rapid detection of singular features in chromosomes [39,40]. Using this method, we showed that the X chromosomes of drosophilid species are less complex than the autosomes and that such a pattern correlates with the acquisition of dosage compensation by neo-X chromosomes [39]. These results suggested that simple sequences may be linked to dosage compensation and that oligonucleotide profiling may be used to detect X chromosome-specific sequences involved in the recognition of that chromosome by the DCC. In this paper, we demonstrate that indeed our method allows for the detection of a peculiar type of sequences, hitherto uncharacterized, which is enriched at the DCC binding regions. We define a repetitive sequence motif that may be involved in X-chromosome detection or in spreading of the DCC along the X chromosome of *Drosophila *species.

## Methods

### Sequences, sequence analyses and genomic data representation

*D. melanogaster *chromosomes (Release 4.3) were obtained from the National Center for Biotechnology Information (NCBI; http://www.ncbi.nlm.nih.gov/). Accession numbers were as follows: X: NC_004354.2; 2L: NT_033779.3; 2R: NT_033778.2; 3L: NT_037436.2; 3R: NT_033777.2. All Blast analyses were also performed online at the corresponding NCBI web page http://blast.ncbi.nlm.nih.gov/Blast.cgi. Figures containing *Drosophila melanogaster *genomic regions were drawn with GBrowse [41].

### Definition of DCC binding regions

For our analyses comparing the DCC binding sites with X chromosome-specific sequences, we used the results obtained by Gilfillan *et al*. ([22]; kindly provided by the authors) as primary data. These data refer to punctual, quantitative signal values in ChIP-chip experiments for multiple probes tested along the X chromosome. We wanted instead to define qualitative DCC binding regions which could be compared with X-specific regions. To do so, we needed to define a minimum signal level and also when two adjacent positive probes could be considered part of the same binding region. After some tests with Gilfillan *et al*. [22] data, we decided to choose a minimum value of signal equal to 2 (i. e., a stringent condition, well above background levels) and a maximum distance between consecutive significant values of 1 Kb to be considered part of the same region. This maximum distance was chosen considering that consecutive oligonucleotides in the tiling arrays are separated in the genome by 50 - 100 nucleotides and that repeats are excluded, leading to significant gaps [21,22].

### Oligonucleotide profiling and definition of X-specific regions

We searched along the X chromosome for regions containing X-specific sequences using oligonucleotide profiling, implemented in our program UVWORD [39,40]. The UVWORD algorithm is simple: the program first reads a selected sequence (source sequence), one nucleotide at a time, and establishes the frequencies of all oligonucleotides of size *k *nucleotides present in that sequence. Then, it reads a second DNA sequence (target sequence), again one nucleotide at a time, and associates each DNA word present in the target sequence with the frequency in the source sequence. By using a single target sequence and two source sequences, relative ratios of frequencies of oligonucleotides in the source sequences can be obtained [39,40]. Here, given that we wanted to establish X-specific regions, the target sequence was the X chromosome and the two source sequences were the X chromosome itself and an autosome, the 2L chromosome arm. In Gallach *et al*. [39], we demonstrated that all *D. melanogaster *major autosomal arms have essentially identical oligonucleotide compositions. Therefore, it is only necessary to use one autosome for comparing with the X chromosome.

The characterization of X-specific regions was therefore performed as follows: for each word of the X chromosome (target), its frequency was determined on the X chromosome (source 1) and on the 2L arm (source 2) and these values were then combined to obtain an X/2L ratio (for words present on the X, but absent on 2L, we fixed a 2L value = 0.5). This X/2L ratio was then corrected for the relative sizes of the X and 2L chromosomes. The final corrected X/2L ratio provides a measure of X-specificity for each word on the X chromosome [39]. However, here our interest was not to establish how X-specific particular words were, but to detect X-specific regions which could be compared to DCC binding sites. Therefore, we decided to average the values of a certain number *R *of adjacent words of size *k *to obtain smoothed profiles of X-specificity. In this study, we used the parameters *k *= 13 and *R *= 5. The reasons for choosing words of 13 nucleotides, which are optimal for *Drosophila *chromosome analyses, are described in Gallach *et al*. [39]. We chose *R *= 5 in order to allow the analysis of small X-specific regions. In summary, the two parameters, *k *= 13 and *R *= 5, define loci consisting on 5 adjacent words of size *k *= 13 of the X chromosome (total size of a locus = 17 nucleotides) for which average X/2L ratios are calculated. Notice that, with each nucleotide that the program reads in the target sequence, a new locus of 17 nucleotides is established, which overlaps *k *+ *R *- 2 = 16 nucleotides with the previous one, and a new average is calculated.

After obtaining the average X/2L ratios, the next step was to establish when a region of the X (i.e., a single locus, or oftentimes, a set of adjacent, overlapping loci) was significantly X-specific. We explored what the effect was of using different threshold values of the average X/2L ratios with respect to their ability to explain DCC binding regions. As shown in the Results section, the best results were obtained with an average X/2L ratio of 6.8 (i. e., when the region contained oligonucleotides that were present on average at least 6.8 times more often on the X than on the 2L arm). Therefore, we eliminated all regions below the 6.8 cutoff and retained the rest for comparison with the DCC binding regions.

### Comparison of DCC binding regions and X-specific regions and characterization of motifs

**Figure 1:**
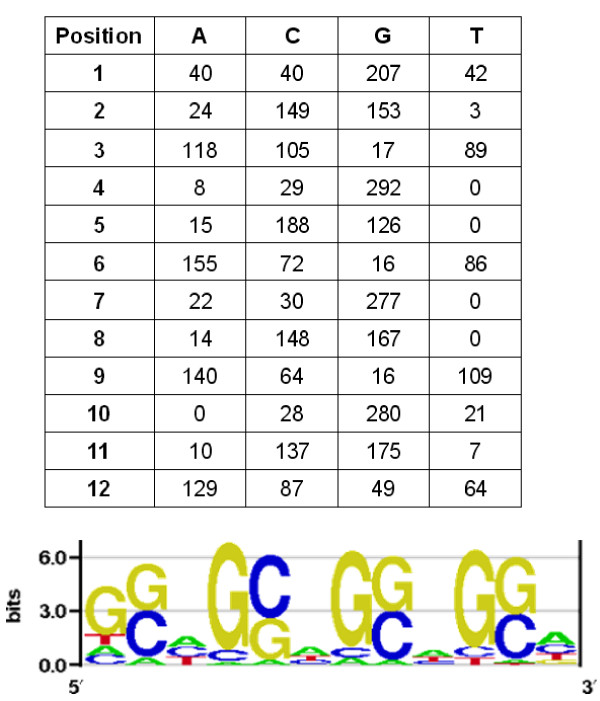
**Frequency matrix of nucleotides and relative entropy-based logo of the detected motif**.

After the DCC binding regions and the X-specific regions were specified, we compared them in order to select a set of overlapping sequences. The selected sequences, which are at the same time able to strongly bind the DCC and significantly X-specific, were analyzed with five of the best available programs designed to detect motifs from sets of sequences. These programs were MEME [42], ALIGNACE [43], WEEDER [44], MOTIFSAMPLER [45] and GLAM2 [46]. The logic for using several programs, with very different algorithms, was to increase the likelihood of finding a set of common motifs in our sequences (see discussion of the programs and strategy in [47-49]). Before using the programs and to avoid biasing their analyses, we eliminated all sequences which were repeated. We checked multiple alternative parameters for each program, and the conditions finally chosen were those which provided the most significant statistical value. When similar scores were obtained with different parameters, we chose the ones that generated the largest number of positive motifs. All sequences which were detected as containing a particular motif in at least four of the five programs were selected for further analysis. We discovered that all of them could be grouped together in a single motif, easily defined using MEME. We then used the applications Prophecy and Profit from EMBOSS [50] to, respectively, generate a frequency matrix from our motifs and to map the motifs along the *D. melanogaster *chromosomes (see raw data in Additional File 1). Average densities and their standard errors were calculated as number of copies of the motif in both DNA chains divided by number of base pairs for 2 Mb-long chromosome fragments. The program enoLOGOS [51] was used to draw the DNA logo shown in Figure 1.

Once the motif enriched in the DCC binding sites based on Gilfillan *et al*. [22] data was characterized, we decided to map it not only against data obtained from that paper, but also against a consensus dataset of DCC binding regions obtained by combining data from Gilfillan *et al*. [22] and Alekseyenko *et al*. [21]. This consensus dataset was obtained as follows: for the three datasets from Alekseyenko *et al*. [21], corresponding to SL2 cells, clone 8 cells and embryos, we characterized regions containing probes with signal values above or equal to 2 in each of the three experiments and separated by at the most 1 Kb (i. e., the same criteria used for the Gilfillan *et al*. [22] dataset in the previous analyses). These regions were then compared to those obtained previously from the Gilfillan *et al*. [22] dataset. When the regions derived from Alekseyenko *et al*. [21] and Gilfillan *et al*. [22] results were separated by less than 1 Kb, they were merged in order to generate the final consensus regions. Given that this way of obtaining the consensus regions led in some cases to the appearance of small regions detected in single experiments, data were filtered, keeping only regions that were longer than 1000 nucleotides.

We checked for the presence of motifs in the autosomal regions shown to be bound by the DCC complex by Gorchakov et al. [36] using similar methods. As above, only the regions with signal values above 2 were considered positive in our analyses of the TrojanElephant transposon. The TrojanHorse sequences were kindly provided by the authors.

### Evolutionary conservation analyses

To determine whether the motifs were evolutionary conserved, the sequence corresponding to each motif plus 100 nucleotides upstream and downstream of it were selected as queries to perform BLASTN searches against *D. virilis *genomic sequences (included in the NCBI wgs database). For those sequences for which non-ambiguous homologies were detected (minimal E-value = 10^-3^, minimal length of homology = 40 nucleotides), we evaluated the percentage of nucleotide identity both within and around the motif. All the sequences for which we found homology in *D. virilis *were reexamined in the *D. melanogaster *genome to determine whether they corresponded to coding regions. The codons that corresponded to the conserved motif sequences included in coding regions were characterized using TBLASTX analyses.

### Coding region analyses

We downloaded the files containing the coding regions of the *D. melanogaster *chromosomes (again, from the 4.3 genome release, obtained from Flybase [52]) and established the frequency of each type of codon. We also determined to which codons the motifs located in coding regions of the X chromosome corresponded, either within or outside the DCC binding regions. Finally, we established the relative position of the motifs along the genes by following methods similar to those described in [22]. Briefly, we obtained from the supplementary material of that paper a list of X chromosome genes with intense DCC binding (average signal > 0.5). From them, we selected those which were larger in size than 2000 base pairs and which also contained at least one motif. For the 421 genes with those features, we mapped all motifs present as follows: the central nucleotide of the motif was found on the X chromosome and a value measuring the relative position along the gene was assigned to that nucleotide (value = [position of the nucleotide on the X chromosome - position of the first (5') nucleotide of the gene on the X chromosome]/total length of the gene). We finally used a Kolmogorov-Smirnov test to establish whether the distribution of motifs was uniform throughout the sequences of the set of genes.

### Analyses of high affinity binding sites

As explained in detail in the Results section, we also established the density of our motif in the high affinity binding sites characterized by Alekseyenko *et al*. [30] and Straub *et al*. [29]. In parallel, we used MEME to reanalyze the data obtained in those two studies in order to establish whether there was any sequence relationship among the motifs detected in the high affinity sites and our motif.

### Sliding-window analyses of motif locations

We determined the nucleotide located at the center of all DCC binding regions (from Gilfillan *et al*. [22] data), in all the HAS (from Straub *et al*. [29] data), and in each region of the X where the DCC does not bind (i. e. the regions that are left along the X chromosome once we eliminate from it the DCC binding regions). We also randomly selected 1000 nucleotides from each large autosomal arm (for a total of 4000 randomly selected points). The central nucleotides of DCC binding regions, non-binding regions, HAS and the randomly taken nucleotides of the autosomes were taken as starting points for sliding-window analyses of motif densities. Those analyses were performed as follows: two 500-nucleotide long windows were initiated at those starting points and then displaced, respectively upstream and downstream from them, one nucleotide at a time. Then, for each window and position, its density of motifs (no. motifs/Mb) was determined. In this way, we obtained a precise characterization of how the density of motifs varies with increasing distances from the starting points.

## Results

### Primary definition of DCC binding regions, X-specific regions and determination of maximal congruence between both types of regions

Our first goal was to unambiguously define DCC binding regions and X-specific regions along the X chromosome and to establish the overlap among them. We started by applying the parameters of signal intensity and proximity between significant probes indicated in the Methods section to the data obtained by Gilfillan *et al*. [22]. We thus defined 559 DCC binding regions. This first result shows that our criteria were quite strict: Gilfillan *et al*. [22] described their data as containing >700 regions of binding. We then defined X-specific regions by checking different cutoff values of X-chromosome specificity, which establishes the enrichment in the X chromosome, with respect to the autosomes, of X chromosome sequences (Table 1). The final X/2L ratio chosen was 6.8, i. e., words in a region had to be at least 6.8 times relatively more abundant on the X than on an autosome to be selected. This value was chosen because it was the one for whose percentage of X-specific regions that overlapped with DCC binding regions was maximal (Table 1; X-positive regions). Thus, we characterized 22366 short X-specific regions, corresponding to 2.3% of the X chromosome. The average size of those regions was 19.6 nucleotides. As indicated also in Table 1, 82% of the DCC binding regions included at least one of the X-specific regions, the average being of 5.4 X-specific regions per DCC binding region.

**Table 1:** Results used in the selection of the cutoff value for X-specificity. In bold, data for the chosen value.

X/2L cutoff (%) ^a^	X regions ^b^	X-positive regions (%) ^c^	DCC positive regions (%) ^d^
14 (0.45)	2267	229 (10.10)	53 (9.48)
12.5 (0.52)	2805	279 (9.94)	77 (13.77)
11 (0.62)	3719	380 (10.22)	112 (20.03)
9.5 (0.81)	5416	551 (10.17)	200 (35.78)
8 (1.28)	10202	1030 (10.10)	326 (58.31)
**6.8 (2.33)**	**22366**	**2475 (11.07)**	**461 (82.47)**
6.1 (3.55)	36798	3948 (10.07)	499 (89.27)

a: Cutoff value for the X/2L ratio and percentage of the X chromosome above that cutoff

b: number of X-specific regions with an X/2L ratio higher than the cutoff

c: number of X-specific regions included in DCC binding regions and percentage respect to the total number of X-specific regions

d: DCC regions including at least one X-specific region. Total number and percentage relative to the 559 DCC binding regions

### Characterization of an X-enriched motif found in DCC binding regions

The X-specific regions included in DCC binding regions (2157, after eliminating duplicates) were analyzed with five motif-recognition programs to determine whether they had anything in common. Notably, each of these five programs indeed detected that many regions had common motifs, although the number of regions detected was quite variable (GLAM2: 1057 regions; MOTIFSAMPLER: 916 regions; MEME: 712 regions; WEEDER: 490 regions; ALIGNACE: 370 regions). We checked for congruence among these results, looking for regions that were detected as significant in at least four of the five programs. This led to the characterization of 329 different sequences. Given that some of them were present more than once in our original dataset, they corresponded to 356 different places along the X chromosome. The expected number of sequences, if the results generated by the programs were independent, was just 102. This difference demonstrated that the five programs often detected the same sequences. Finally, we used MEME to analyze the 329 different sequences obtained, establishing that all of them truly contained a single 12-nucleotide long motif. According to MEME, the probability of such a motif arising by chance so many times in that sample was 10^-523^. In related analyses, we found that X-specific regions not included in the DCC binding regions are just slightly enriched in simple DNA sequences, mainly poly-G/C [53]. These results demonstrate that there is a common motif in at least 356 places of the X-chromosome that are both highly X-specific (X/2L ratio ≥ 6.8) and where the DCC binds. The motif obtained is shown in Figure 1. It has an obvious repetitive signature: [G(CG)N/N(CG)C]_4_. From now on, we will refer to this motif as [G(CG)N]_4_.

### Chromosomal distribution of sequences related to the motif

In order to determine all the positions in which sequences related to the [G(CG)N]_4 _motif were present, we used the Prophecy and Profit programs of the EMBOSS suite. We established the maximum score (= sum of the frequencies of the most frequent nucleotides) for the matrix obtained for our motif from the 329 sequences characterized (Figure 1), and then we determined all the sequences with a score of at least 90% of that maximum. We then scanned the X chromosome for the positions of all the sequences with score values above the 90% cutoff. This led to the characterization of 14967 sites. These sites had an average size of 14.4 nucleotides, slightly larger than the original 12 nucleotides-long motif. This was caused by the fact that the motif is internally repetitive, so positive overlapping sequences were often detected and merged.

We found that 3082 of the 14967 sites were included in 449 of the 559 DCC binding regions defined from Gilfillan *et al*. [22] data, with an average of 6.9 sites/DCC region. It is significant that, although we detected sites in just 80% of the DCC binding regions, the ones that remained undetected were in general very small. The total length of the regions lacking motifs was just 6% of the total assigned to DCC binding regions. We then divided the X chromosome into the 559 DDC binding regions and the rest, in order to test whether the sites detected were found at a higher density in those regions. For the rest of the X chromosome, once the DCC binding regions were excluded, we determined an average of 602 ± 24 sites/Mb, a value almost identical to the one that we found for the autosomes: 590 ± 29 sites/Mb (all large autosomal arms considered). However, the 449 positive regions had a density of 1582 ± 68 sites/Mb, that is, almost three times higher. Logically, this leads to these sites being much closer in the DCC binding regions than in the rest of the chromosomes (Additional File 2). The median distance between consecutive sites was just 215 bp and 87% of the consecutive sites were separated by less than 1 Kb. These results demonstrate that most DCC binding regions contain multiple sites related to the [G(CG)N]_4 _motif that we detected in our original searches, that those sites are relatively close to each other and that they are significantly enriched in most DCC binding regions, with respect to both the rest of the X chromosome and the autosomes.

To obtain further support for these conclusions, we repeated these analyses with a consensus dataset, generated combining data from Gilfillan *et al*. ([22]; 1 dataset) and Alekseyenko *et al*. ([21]; 3 datasets). From the combined data, we characterized 666 DCC binding regions. These are quite more than in our analysis based on a single dataset (666 vs. 559), which suggests that the four datasets were somewhat heterogeneous. Using again the 90% of maximum value of the similarity matrix as a cutoff, sequences related to our motif were found in 79% (526/666) of these consensus DCC binding regions. As previously seen, the DCC binding regions in which the motif was not detected were small, corresponding all together to just 4% of all the nucleotides defined as bound by the DCC in the consensus dataset. The density of motifs was 1201 ± 39 sites/Mb in the positive DCC binding regions in this consensus dataset. The density in the rest of the X chromosome went down to 523 ± 25 sites/Mb in this case, thus being a bit lower than the average 590 sites/Mb detected in the autosomes. These results confirmed the significant enrichment of the motif in the DCC binding regions.

**Figure 2:**
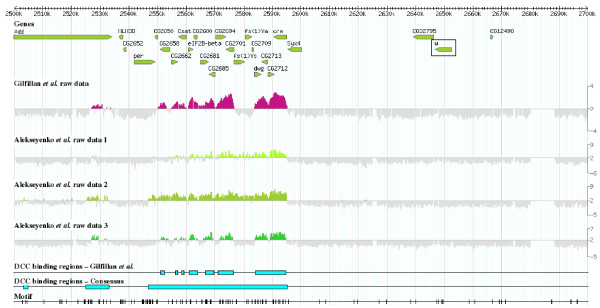
**Location of the [G(CG)N]_4 _motif along the region that contains the *white *gene (*w*, rectangle)**. The figure includes the coordinates in the X chromosome, location of the genes, raw data of binding obtained from Gilfillan *et al*. [22] and Alekseyenko *et al*. [21] experiments, the binding regions derived from Gilfillan *et al*. [22], the consensus binding regions and the precise positions of the motifs. Notice the clear difference in motif densities between the left part of the figure, where the DCC binding regions are located, and the right part, in which DCC binding regions have not been found.

Figure 2 shows a typical example of how motifs are distributed. The figure shows a 200 Kb region that contains the *white *gene, half of which exhibits strong DCC binding while the other half does not. The high concentration of motifs around the DCC binding regions upstream of *white *is evident, while the other half unbound by the DCC, which includes the *white *gene itself, shows a significantly lower number of motifs.

We also checked for the presence of our motifs in the TrojanHorse and TrojanElephant transposons used by Gorchakov et al. [36] to demonstrate the ability of some autosomal sequences to acquire *de novo *DCC binding. Significantly, we found that the region in TrojanHorse to which the DCC binds when the transposon is inserted on the X chromosome (corresponding to positions 4224822-4228454 in chromosome 2L; genes *CG3702 *and *Rpl40*) has a high motif density (4 sites in 3633 nucleotides, or 1101 sites/Mb) while the rest of the transposon has a low density (9 sites in 19531 nucleotides; 461 sites/Mb). Similarly, the regions bound by the DCC in the TrojanElephant transposon (signal > 2; corresponding to positions 6908636-6914771 and 6949036-6955236 in chromosome 2L) have a density of 811 sites/Mb (10 motifs in 12337 nucleotides) while the rest of the transposon has a density of motifs of just 623 sites/Mb. In summary, the density of sites in the regions bound by the DCC when these transposons are localized on the X chromosome is much higher than the average density of sites in the autosomes (590 sites/Mb). This may contribute to them behaving as typical X chromosome sequences when inserted on the X.

### Evolutionary conservation of the sites located in DCC regions

The DCC is acting not only in *D. melanogaster*, but also in other drosophilids, including flies of genera other than *Drosophila *[54]. Therefore, we decided to explore whether the sites found were evolutionary conserved, which would indicate that the same sequences might be used in different species. Given that the sites are very small, we decided to locate each site in *D. melanogaster *and then add at both sides of the sites a total of 100 extra nucleotides. By doing so, we increased the likelihood of finding the homologous site in a different species. The chosen species to compare was *D. virilis*, which is a distant relative of *D. melanogaster*, belonging to a different subgenus and which genome has been fully sequenced [55]. The two lineages split about 63 millions of years ago [56].

Of the 3082 *D. melanogaster *sites tested, we were able to characterize the homologous *D. virilis *sites in 894 cases. The total number of aligned nucleotides was 113225, of which 12214 corresponded to the sites and the rest to the adjacent sequences added to perform the analyses. Since we were interested in determining whether the sites were evolutionary conserved, we counted the number of differences in both the sites and the adjacent sequences, which was used as a control of local conservation in *D. virilis*. In total, there were 16365 nucleotide differences between *D. melanogaster *and *D. virilis*, and 1448 of them affected the sites. This means that the percentage of nucleotide identity between the sequences of the two species was 88.1% for the sites and just 85.2% for the adjacent sequences. We used a cumulative hypergeometric distribution to establish the probability of the sites and adjacent sequences having the same mutation rate. That probability was 5.0 10^-19^. We conclude that, in the cases in which it has been possible to establish homology between *D. melanogaster *and *D. virilis*, the sites are significantly more conserved than their immediately adjacent sequences.

When we checked for the positions of the 894 conserved sites in the *D. melanogaster *genome, we found that in 781 cases (87%), the motifs were part of the coding regions. We examined those 781 motifs to determine to which codons they corresponded. The rationale was to establish whether the [G(CG)N]_4 _DNA signature generated singular amino acidic signatures. The degenerate [G(CG)N]_4 _pattern can be converted, depending on the frame used, into six codon classes, each one of them corresponding to eight different codons (Table 2). One of these types of codons, which can be summarized in the sequence [(CG)NG]_n_, was found much more often that the rest, while several others were very infrequent (Table 2). Notably, there was a clear correlation between the potential different number of encoded amino acids and the frequency in which the codons were detected. Thus, the preferred (CG)NG triplet is the only one among the six possible classes of codons that encodes for eight different amino acids, while the two less frequent triplets, G(CG)N and (CG)CN, are the only ones that encode for just two amino acids (Table 2).

**Table 2:** Frequency of appearance in the conserved regions analyzed in *D. melanogaster *and *D. virilis *of the six possible codon classes that can be obtained from the repetitive [G(CG)N]_4 _signature

Codon class	Frequency	Amino acids encoded
[(CG)NG]_n_	495	Val, Ala, Glu, Gly, Leu, Pro, Gln, Arg
[CN(CG)]_n_	123	Leu x 2, Pro x 2, Arg x 2, His, Gln
[N(CG)C]_n_	62	Ser x 2, Cys, Pro, Arg, Thr, Ala, Gly
[NG(CG)]_n_	39	Arg x 3, Gly x 2, Cys, Trp, Ser
[G(CG)N]_n_	36	Ala x 4, Gly x 4
[(CG)CN]_n_	26	Pro x 4, Ala x 4

For each codon class, the [XXX]_n _nomenclature refers to the *n *consecutive codons of the same class produced by the DNA repeat. Given the eight-fold degeneracy of that pattern, the set of codons in each codon class may encode for at most 8 amino acids. The amino acids that each codon class encode are also indicated. Numbers after the x sign indicate how many different codons encode for a same amino acid, when they are more than one.

### Coding regions analyses

The results detailed in the previous section suggest a preference for (CG)NG codons to be included in the motifs found in the coding regions bound by the DCC. However, those results were constrained to the 894 cases for which we detected homology with *D. virilis*. Therefore, we decided to establish whether that was a general preference, for all the *D. melanogaster *sites. We found that a total of 2720 of the 3082 sites detected in our previous analyses (88%) were included in coding regions. This is in agreement with the known enrichment of the DCC binding regions in coding sequences [21,22]. Then, we determined that those 2720 sites corresponded to 10701 codons (incomplete codons were discarded). Just as it occurred with the conserved sequences described above, the most frequent triplet was again (CG)NG (6185 codons; 58%). When we performed the same analyses for the motifs not included in the DCC binding regions, we found that 5767 of the 11885 motifs (49%) were included in coding regions. We established that 24016 codons were derived from those 5767 motifs. Notably, the proportion of (CG)NG triplets was quite smaller than in DCC regions, just at 51%. This difference is statistically highly significant (p = 1.2 10^-41^; Chi-square test with 1 degree of freedom). Therefore, the DCC binding regions are enriched in motifs that correspond to (CG)NG codons.

Interestingly, seven of the eight amino acids that the (CG)NG sequences can generate are among the preferred ones in *D. melanogaster *and many other *Drosophila *species [57]. It is known that the *D. melanogaster *X chromosome has an increased codon bias respect to the autosomes [58]. Therefore, it is possible that selective pressure to keep DCC binding/spreading sites with [(CG)NG]_n _signatures contribute, along with other processes, to that X-specific bias. On the other hand, it is important to demonstrate that the opposite is not true (i. e. that our results are not simply caused by the difference in codon bias between the X chromosome and the autosomes). Given that the DCC binding regions and coding regions are often coincidental, a very strong codon bias, or indeed any force causing highly biased frequencies of particular codons on the X respect to the autosomes, could often lead to recovering X-specific codons when using the oligonucleotide profile method to detect X-specific sequences. However, we have found that the frequencies of all codons are actually so similar in the X chromosome and the autosomes that they cannot explain the 6.8× level of enrichment for sequences with *k *= 13 that we have used as cutoff in our searches. When we analyzed the coding regions of X chromosome and autosomes, we found that the codon GGC was the one with the highest X/A relative ratio, which had a value of 1.18. Therefore, even a sequence formed by six consecutive GGC codons (remember that 19.6 nucleotides is the average sequence size selected by our method, see above) would be enriched just 1.18^6 ^= 2.7 times due to its increased presence in the coding regions of the X chromosome respect to the coding regions of the autosomes. It can be deduced that, unless those same sequences are (obviously by reasons totally independent to codon bias or any other forces acting on coding regions) also depleted from the non-coding regions of the autosomes relative to the non-coding regions of the X chromosome, they would never achieve the 6.8× cutoff value required to be detected in our searches. As a relevant example to ascertain this point, we established the relative frequencies of [(CG)NG]_4 _sequences in X chromosome and in autosome genes. These frequencies were 5.5 10^-3 ^(i. e. just 5.5 out of 1000 groups of four consecutive codons had a [(CG)NG]_4 _signature) and 3.7 10^-3 ^respectively, with an X/A ratio of just 1.5. We conclude that neither codon bias nor any other force acting solely on coding regions can explain the selection of these sequences by the oligonucleotide profile searches.

Given that it has been described that the DCC binding regions are preferentially located at the 3' ends of the genes, we explored whether the motifs were similarly distributed (see Methods). We found a depletion of motifs at both extremes of the genes, spanning about the first 5% and the last 5% of their sequences. However, along the rest of the sequences of the genes, the distribution of motifs was homogeneous, according to a Kolmogorov-Smirnov test for departures of the uniform distribution (p = 0.09; n. s.). Thus, the motifs by themselves cannot explain the preference of the DCC to bind the 3' ends of the genes.

### Analyses of DCC high-affinity sites

A relevant point was to determine whether the [G(CG)N]_4 _motif found was in some way involved in the DCC high-affinity sites (HAS). Alekseyenko *et al*. [30] recently characterized a motif, which they called "MSL recognition element" or MRE. This motif was deduced from 150 binding sites detected for the incomplete DCC complex formed in mutant *msl-3 *flies. These 150 sites were considered HAS by those authors. As we already mentioned, this 21 bp motif is characterized by containing (GA/TC)_n _repeats and is totally unrelated to the one that we found. However, using MEME, we detected three other motifs enriched in the putative HAS characterized by Alekseyenko *et al*. ([30]; kindly provided by the authors). Most interestingly, one of the motifs, found a total of 166 times in 79 of their 150 HAS, was clearly related with the motif detected in the searches described above. This motif detected by MEME was 21 base-pairs long and had a consensus sequence which can be simplified to [G(CG)(AT)]_7_. This is obviously very similar to the [G(CG)N]_4 _signature detected in this study. In fact, when we searched for our 12 bp-long motif in the 150 HAS (again using a 90% cutoff for the similarity matrix, as above), we found similar data: 71 HAS contained a total of 138 copies of the motif. Density in the positive HAS was 1004 sites/Mb, slightly lower than that found for the whole set of DCC binding regions, but still higher than that found in the rest of the X chromosome or the autosomes.

In another analysis, Straub *et al*. [29] also searched for HAS, characterized this time by a combination of experiments in cell lines, using either RNA interference of genes encoding DCC proteins (*msl-3*, *mle*, *mof*) or decrease of crosslinking in the ChIP experiments. They obtained a total of 132 putative HAS, from which they characterized a 29 bp motif with (GA/TC)_n _repeats, clearly related to the one found by Alekseyenko *et al*. [30]. Our own reanalysis of those 132 putative HAS using MEME led to the characterization of a total of five motifs, of which the most abundant was roughly equivalent to, although slightly shorter than, the one described by Straub *et al*. [29] (data not shown). None of those five motifs was clearly related to the one we characterized. However, when we searched for our [G(CG)N]_4 _motif in the 132 putative HAS characterized by Straub *et al*. [29], we found that 56 of them actually contain at least one, making a total of 109 motifs. This corresponds to a density in the positive HAS of 1354 motifs/Mb, similar to that found for all the DCC binding sites together and again higher than that found for the rest of the X chromosome and the autosomes (see above). We can conclude from our analyses of Alekseyenko *et al*. [30] and Straub *et al*. [29] data that the [G(CG)N]_4 _motif that we have detected may be contributing to some of the high affinity sites.

**Figure 3:**
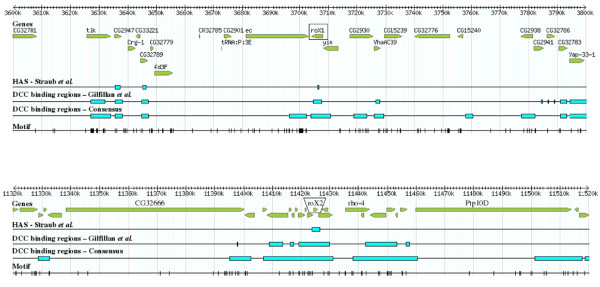
**Distribution of the motif along the regions that contain *roX1 *(top) and *roX2 *(bottom; the precise positions of the two genes are highlighted)**. This figure is similar to Figure 2, except that it also includes the positions of the HAS characterized by Straub *et al*. [29]. The *roX1 *and *roX2 *loci contain a HAS but lack motifs, even although there are many of them in adjacent regions.

We also checked whether the [G(CG)N]_4 _motif was found in individually characterized HAS, which have been determined with a higher precision than that provided by the ChIP-chip experiments. We analyzed the HAS in *roX1 *(217 bp; ref. [59]), *roX2 *(110 bp; ref. [60]), the 18D10 region (510 bp; ref. [61]), the 8F7 region (DBF6; 25 bp), the 18D3 region (DBF9; 59 bp) and the 11B13 region (DBF12; 40 bp; these last three characterized by Dahlsveen *et al*. [24] and Gilfillan *et al*. [25]). We found that only one of them, the one located in the 18D10 region, contained a copy of the motif (again using the 90% similarity matrix cutoff). Actually, the *roX1 *and *roX2 *loci, which may be acting as nucleation centers for DCC formation [62], lack motifs, although they are surrounded by "low affinity" DCC binding regions with a high density of them (Figure 3). We conclude from all these results that the [G(CG)N]_4 _motif may be relevant but is not essential to form the HAS for the dosage compensation complex.

**Figure 4:**
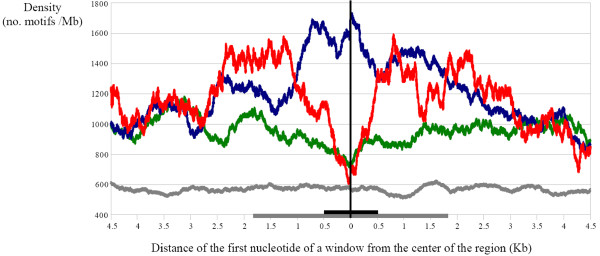
**Average densities of the motifs in different regions**. Densities are measured as no. motifs/Mb in a window of 500 nucleotides. Averages were calculated for all DCC-binding regions (blue), HAS (red) and regions not bound by the complex on the X chromosome (green) and for 4000 randomly sampled regions of the autosomes (1000 per major autosomal arm; in grey). The 500 nucleotides-long windows were slided (one nucleotide at a time and in both directions) from the center of the regions (position indicated as "0" in the X axis), to determine how densities vary when we move away from that center. The broad horizontal grey and black lines indicate the average length of DCC-binding regions and HAS, respectively. All analyses were oriented respect to the positions of the telomeres and centromeres of the chromosomes. Thus, values at the left of the "0" position are obtained when the windows are moved towards the telomere and those at the right, when they are slided towards the centromere.

We then performed a sliding-window analyses of the average density of motifs along the X chromosome, both in regions not bound by the DCC, in the DCC-binding regions and in the regions corresponding to HAS. The nucleotides at the middle of each of those regions were taken as starting points to determine the average density in windows of 500 nucleotides. Those windows were slided, one nucleotide at a time and in both directions, to determine how density varied with progressive distance from the center of the region. Autosomes were used as negative controls. A total of 1000 random nucleotides per major autosomal arm were sampled and, from them, the sliding process repeated. Results are shown in Figure 4. Differences between DCC binding regions and HAS are striking. In the DCC binding regions, average density is maximum at their centers and progressively declines to reach X-specific background levels (i. e., the level found if we start the analysis from a region of the X chromosome not bound by the DCC) at about 2.5 - 3 Kb at each side of the center of the region. This is reasonable given that the average size of the DCC binding regions is about 3.8 Kb (Figure 4): once we abandon the DCC binding regions, we expect the density to substantially diminish. On the contrary, and surprisingly, the centers of the HAS have a minimum number of motifs, which is equivalent to that observed at the center of the regions not bound by the DCC (Figure 4). Density then increases to reach maximum levels at about 0.5 - 1 Kb from the center, i. e., approximately at both ends of an average-sized HAS. After we further move away from the center of the HAS, density decreases again, to reach again X-specific background levels at about 3 Kbs from the middle point of the HAS. These results demonstrate that the motifs are placed differently in HAS and DCC binding regions. The increase at the ends of the HAS and posterior decrease when we move away from the end of the HAS can be understood considering the fact that HAS are commonly included within larger "low affinity" DCC binding regions, which have many motifs (see cases in Figure 3). Therefore, a simple explanation is that the frequency of the motifs increases when we move from the center of the HAS towards the "low affinity" DCC binding regions that surround the HAS. A final significant detail is that both the progressive increase in density when we get away from the center of regions of the X chromosome not bound by the complex and the fact that those non-bound regions have an average density of motifs which is higher than that found in the autosomes (a result that apparently contradicts the identical average densities mentioned above, but can be easily observed in Figure 4) are due to the interspersion of those regions with the motif-rich DCC binding regions. Once we move along the X chromosome from the center of the non-bound regions, we come upon DCC binding regions, leading to a subsequent increase of average density.

## Discussion

We have characterized a repetitive sequence motif, [G(CG)N]_4_, which is specifically enriched in regions bound by the *Drosophila *DCC. Significantly, hexanucleotides related to the motif that we have described had been detected already as enriched in DCC binding regions [22]. Our ability to detect this longer motif critically depended on a new type of approach, which involved not only taking into account the similarities among DCC binding regions but also the local X-specificity of the sequences within those regions. In this sense, our application of the oligonucleotide profiling strategy, which previously demonstrated its power to tackle more general problems of chromosome differentiation [39,40], has been fruitful. Further applications of this approach to related problems can be easily envisaged.

The motif is especially enriched in the DCC binding regions that do not have a high affinity for the complex (i. e. those that are not part of the HAS determined so far), while is less frequent in the HAS, in which, moreover, the motifs mostly appear in peripheral positions (see statistical results and Figure 4). This distribution suggests a significant role in the generation of most "low affinity" DCC binding regions, including those that surround the HAS. Its significance within the HAS is less obvious; it may simply contribute to them in some cases. This is demonstrated by the facts that many HAS lack motifs, while the motif tends to be at high densities adjacent to the HAS (see figures 2, 3 and 4 and numerical results above). It is significant in this context that the motifs found here may also contribute to explain recent results showing acquisition of DCC binding sites by some short, autosome-derived regions transposed to the X chromosome [36]. The failure of acquiring DCC binding by larger regions of autosomal origin [28] may be simply understood considering that the larger the region, the closer its density of motifs will be to the autosomal average, which may be too low to support binding. Thus, our hypothesis to explain Gorchakov et al. data [36] is that only particular, short, motif-rich regions of autosomal origin may acquire dosage compensation when moved to the X chromosome. The active transcription of genes located in those regions seems also essential, given that a deletion in TrojanHorse that abolishes DCC binding [36] does not eliminate any [G(CG)N]_4 _motif.

With these results in mind, we hypothesize that the [G(CG)N]_4 _sequences may contribute to the recognition of the X chromosome by the DCC, to generate the majority of DCC binding regions observed, and/or to the spreading of the complex from the HAS to the rest of the X chromosome. Our results are in agreement with the combined model that we discussed in the Introduction, according to which there are multiple binding/spreading sites with different features and strengths along the X chromosome. The available data is compatible with low affinity sites being largely explained by the presence of multiple, closely located [G(CG)N]_4 _sequences, while high affinity sites may be due to a specific combination of sequence motifs among which the (GA/TC)_n _motif would be fundamental and the [G(CG)N]_4 _motif and probably several others (e. g. those additional kinds of sequences detected in our MEME searches as enriched at HAS) of secondary importance. Our data thus indicate that the high affinity sites indeed are qualitatively different from the rest in terms of which sequences are involved in their formation, as already suggested by Straub *et al*. [29] and Alekseyenko *et al*. [30] data.

The fact that some small DCC binding regions (which add up to 4 - 6% of all nucleotides bound by the DCC) do not contain any obvious motif, can be explained in different ways. First, it is evident that our analyses do not exclude that other motifs, so far uncharacterized, may be significant in DCC binding regions. Our searches have been quite stringent and focused on motifs of a certain length (in the range of 10 - 20 nucleotides, given the *k *and *R *parameters used), so some regularities may have been missed. Alternatively, it is possible that the criterion used to search for motif sequences (90% of the maximum score of the frequency matrix) is too strict. Finally, these exceptional regions may be simply caused by experimental limitations (e. g., false positives for DCC binding).

Despite that it was often impossible to establish whether particular [G(CG)N]_4 _motifs within DCC binding regions were evolutionary conserved, we were able to detect 894 sites in *D. virilis*. We found that 87% (781/894) of them were included in coding regions. The higher level of conservation of the [G(CG)N]_4 _motif with respect to the immediately adjacent sequences detected in those 781 cases is significant. It may be interpreted as [G(CG)N]_4 _sequences being part of the X-recognition/DCC spreading system in multiple *Drosophila *genus species, a result that is in agreement with the known conservation of DCC binding in drosophilids [50]. In Gallach *et al*. [37], we described the most frequent trinucleotides in different *Drosophila *species. Interestingly, one of them was (CAG/CTG)_n_, which corresponds to one of the possible trinucleotides defined by the [G(CG)N]_4 _motif. This trinucleotide was enriched on the X chromosomes of all *Drosophila *species tested, including the neo-X arm of *D. pseudoobscura *[37]. These results, together with those found for the high affinity sites, enriched for (GA/TC)_n _sequences, offer support to the old idea that different types of simple DNA along the *Drosophila *X chromosome may be key to the process of dosage compensation (see discussions in refs. [39,63-65]). It is interesting that Bachtrog [66] recently characterized positive selection acting on three HAS in the *D. melanogaster *lineage, a result that correlates with positive selection of MSL proteins in that same lineage [67,68]. These results, which contrast with the evolutionary conservation that we have observed for our motif in the *D. melanogaster/D. virilis *comparisons, suggest that HAS may be, at least in particular lineages, under peculiar selective regimes, different from the rest of DCC binding regions, as suggested by Bachtrog [66].

When we established the imprint that the [G(CG)N]_4 _motif may leave on the protein sequences of *Drosophila *genes, we found that the preferred frame was the one that allowed for the maximum variation in the amino acids encoded by the nucleotides of the motif. This result may contribute to explain the apparent paradox of why the DCC often binds to coding regions of the genes [21,22]. The adjacent non-coding regions, evolutionary less constrained, would seem better targets to acquire binding sites for the DCC (see ref. [65] for a discussion of the process of acquisition of dosage compensation). However, simple, degenerate, repetitive motifs have a minimal impact on coding regions if they are able to encode for many different amino acids. Then, it may be often enough to choose particular codons to generate the DNA motif without changing the protein sequence. Only if the motif encodes for just a few amino acids, its generation would necessarily cause a repeat of those amino acids in the protein, which may be more difficult to accommodate. In the case of the motif that we discovered, the most versatile frame, and the one that is found most often, is [(CG)NG]_n_, which can be converted into eight different amino acids (Table 2). As we indicated above, the acquisition of these sequences may contribute to codon bias, which is especially significant in the X chromosome [55].

## Conclusions

The analysis of the Chip-ChIP data for DCC binding with a novel strategy based on oligonucleotide profiling allowed us to detect a set of DNA sequences that are at the same time X-specific and included in the DCC binding regions. These sequences share a common short, internally repetitive motif which may contribute to the establishment of the wild-type localization of the dosage compensation regulators along the X chromosome. Further experimental confirmation for the role of the motif detected in DCC binding and/or local spreading is required. These results open fascinating new possibilities. Among them, the clearest is that it is now possible to devise experiments to test more precisely the factors governing the binding or spreading of the DCC complex. Also, the relationships among genetic changes and epigenetic modifications leading to the wild-type pattern of DCC binding may be explored in more detail. Finally, we also may more deeply understand the similarities and differences of the evolution of the dosage compensation systems of *Drosophila*, *Caenorhabditis*, in which a similar situation of simple, short motifs acting as X chromosome recognition/spreading marks has been described [69-71], and mammals, in which several types of repetitive DNA sequences may influence X chromosome inactivation [72,73].

## Authors' contributions

MG contributed to the design and performed most of the analyses described in the text. He also significantly contributed to the ideas developed in the manuscript. VA generated the programs required to implement the oligonucleotide profiling strategy. RA generated the programs and obtained the results described in the "Coding region analyses" sections. IM devised and supervised the research and wrote the manuscript. All authors read and approved the final manuscript.

## Supplementary Material

Additional file 1 **Tables S1 - S3**. Tables with the coordinates on X chromosome for the DCC binding regions, the 2475 X positive regions and the copies of the motif. Click here for file

Additional file 2 Cumulative distribution of the proximity among consecutive sites. Distances are measured in hundreds of nucleotides. Continuous black line: sites on the DCC binding regions. Continuous grey line: X chromosome. Dotted black line: X chromosome, outside of the DCC binding regions. Dotted grey line: autosomes. Microsoft Word (.doc) file. Click here for file
