# Supplementary figures and images for: A sequence motif enriched in regions bound by the Drosophila dosage compensation complex
Source: BMC Genomics. 2010 Mar 12;11:169. doi: 10.1186/1471-2164-11-169 (PMC2848247; doi:10.1186/1471-2164-11-169)

**Figure S1. Gallach et al.**


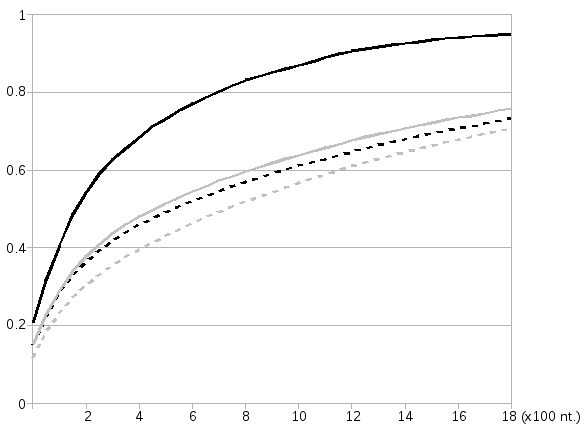

Supplement: Additional file 2 — Cumulative distribution of the proximity among consecutive sites. Distances are measured in hundreds of nucleotides. Continuous black line: sites on the DCC binding regions. Continuous grey line: X chromosome. Dotted black line: X chromosome, outside of the DCC binding regions. Dotted grey line: autosomes. Microsoft Word (.doc) file. [file 1471-2164-11-169-S2.DOC]
